# Supplementary material for: Sexual dimorphism and allometry in the sphecophilous rove beetle Triacrus dilatus
Source: PeerJ. 2015 Jul 28;3:e1123. doi: 10.7717/peerj.1123 (PMC4525698; doi:10.7717/peerj.1123)
Supplement: Table S2 [file peerj-03-1123-s008.docx]

Supplemental Table S2. Results from the Shapiro-Wilk test and Levene’s test for equal variances for each variable and gender. N=29 males; N=22 females.

|  | | **Elytra Length** | | **Left Mandible** | | | **Ocular Distance** | |
| --- | --- | --- | --- | --- | --- | --- | --- | --- |
|  |  | ***Male*** | ***Female*** | ***Male*** | | ***Female*** | ***Male*** | ***Female*** |
| **Shapiro-Wilk Test for Normality** | **W statistic** | 0.970 | 0.942 | 0.974 | 0.968 | | 0.956 | 0.963 |
|  | **p-value** | 0.569 | 0.213 | 0.678 | 0.663 | | 0.261 | 0.544 |
| **Levene’s Test for Equal Variances** | **F statistic** | 0.626 | | 0.282 | | | 0.924 | |
|  | **p-value** | 0.432 | | 0.598 | | | 0.341 | |
