# Supplementary material for: Sexual dimorphism and allometry in the sphecophilous rove beetle Triacrus dilatus
Source: PeerJ. 2015 Jul 28;3:e1123. doi: 10.7717/peerj.1123 (PMC4525698; doi:10.7717/peerj.1123)
Supplement: Table S3 — N, sample size; Mean, mean value for each variable; SD, standard deviation; SE, standard error; CV, coefficient of variation (%). * Means were significantly different between males and females of that variable using a 2-tailed t-test, p < 0.05. [file peerj-03-1123-s009.docx]

Supplemental Table S3. Summary statistics and coefficient of variations for each variable and gender. N = sample size; Mean = mean value for each variable; SD = standard deviation; SE = standard error; CV = coefficient of variation (%). * Means were significantly different between males and females of that variable using a 2-tailed t-test, p<0.05.

|  | **Elytra Length** | | **Left Mandible *** | | **Ocular Distance *** | |
| --- | --- | --- | --- | --- | --- | --- |
|  | ***Male*** | ***Female*** | ***Male*** | ***Female*** | ***Male*** | ***Female*** |
| **N** | 29 | 22 | 29 | 22 | 29 | 22 |
| **Mean** | 4.32 | 4.15 | 4.17 | 3.57 | 3.76 | 3.39 |
| **SD** | 0.350 | 0.397 | 0.843 | 0.761 | 0.566 | 0.469 |
| **SE** | 0.065 | 0.085 | 0.157 | 0.162 | 0.105 | 0.10 |
| **CV (%)** | 8.09 | 9.55 | 20.19 | 21.32 | 15.04 | 13.84 |
